# Elevated ICAM5 as a promising predictor of poor prognosis in bladder cancer via EMT, immune microenvironment, and therapy resistance

**DOI:** 10.1371/journal.pone.0347623

**Published:** 2026-06-08

**Authors:** Xiaolong Chen, Zhen Wang, Yangsheng Rong, Zhiqiang Zhu, Zheng Peng, Kunyuan Huang, Guanyun Deng, Di Liu, Qing Wang, Jianguo Zhu, Kun Chen, Kehua Jiang

**Affiliations:** 1 Department of Urology, Guizhou Provincial People’s Hospital, Guiyang, Guizhou, China; 2 Department of Urology, Weng’an County People’s Hospital, Guizhou, China; 3 Guizhou Medical University, Guiyang, Guizhou, China; 4 Department of Urology, People’s Hospital of Qiandongnan Miao and Dong Autonomous Prefecture, Kaili; 5 Department of Medical Genetics, Guizhou Provincial People’s Hospital, Guiyang, China; Shanghai Jiao Tong University, CHINA

## Abstract

**Background:**

Bladder cancer, the most common malignancy of the urinary system, is associated with poor prognosis due to its metastatic potential, invasive behavior, and immune evasion. Intercellular adhesion molecule 5 (ICAM5), a member of the immunoglobulin superfamily, regulates cell adhesion and has been implicated in tumor progression. However, its biological function in bladder cancer remains unclear.

**Methods:**

In this study, we analyzed data from The Cancer Genome Atlas (TCGA) and UCSC Xena databases to investigate ICAM5 expression, prognostic significance, genetic mutations, methylation, immune profiles, and regulatory functions in bladder cancer. Weighted Gene Coexpression Network Analysis (WGCNA) and Gene Set Cancer Analysis (GSCA) were employed to explore ICAM5-related pathways.

**Results:**

Our findings demonstrated that ICAM5 expression was significantly upregulated in bladder cancer and associated with advanced disease features, including higher TNM stages, pathological grades, and aggressive molecular subtypes. Furthermore, ICAM5 influenced the immune microenvironment, regulated methylation, and modulated immune checkpoint expression, contributing to immunotherapy resistance. Mechanistically, ICAM5 promoted epithelial-mesenchymal transition (EMT), proliferation, and metastasis.

**Conclusions:**

ICAM5 serves as a novel prognostic biomarker and potential therapeutic target in bladder cancer, orchestrating EMT progression, reshaping the immune microenvironment, and driving resistance to immunotherapy.

## 1. Introduction

Bladder cancer (BCa) is one of the most common urinary system malignancies because of its high recurrence and progression rates. Approximately 75% of newly diagnosed bladder cancers are classified as nonmuscle invasive bladder cancer (NMIBC) [1,2]. The recommended treatment for NMIBC is transurethral resection of the bladder tumor. However, BCa has a high recurrence rate, and approximately 25% of patients with NMIBC are more likely to develop muscle-invasive tumors despite receiving treatment [3,4]. In recent years, numerous studies have been conducted to improve our understanding and develop promising clinical approaches for the treatment of BCa. Innovative cystoscopy techniques, including optical and imaging equipment, as well as tumor biomarker-based noninvasive urine screening tools like DNA methylation testing, have significantly improved the accuracy of tumor detection. This, in turn, has decreased the risk of bladder cancer recurrence and progression. However, Biomarkers for BCa diagnosis are improperly precise and provide uncertain clinical advantages for individuals with early low-grade BCa. In addition, biomarker levels may differ depending on the functionality of organs and drugs.Thus, reliable prognostic markers and therapeutic targets for bladder cancer are urgently needed.

ICAM5, an intercellular adhesion molecule, has been implicated in tumorigenesis and perineural invasion [5]. Upregulated ICAM5 expression has been identified as a risk factor for poor survival in glioblastoma [6], and its overexpression in colon cancer further supports its potential as a diagnostic and prognostic biomarker across malignancies. However, the specific role and clinical relevance of ICAM5 in bladder cancer remain largely unexplored. In this study, we observed elevated ICAM5 expression in bladder cancer tissues, correlating with enhanced cancer cell motility and invasion. Therefore, the objectives of this study were (1) to systematically characterize the expression pattern and clinical significance of ICAM5 in bladder cancer, (2) to investigate its functional role in tumor progression, and (3) to assess its prognostic value as a potential biomarker. Through integrated bioinformatic analysis and experimental validation, we aimed to provide a comprehensive understanding of ICAM5 in bladder cancer biology and its translational potential.

## 2. Materials and methods

### Date collection and preprocessing

To ensure the reproducibility and methodological rigor of our study, we provide a detailed description of the data collection and preprocessing steps. Normalized gene expression profiles (RNA-Seq by Expectation-Maximization [RSEM] values), tumor mutation burden (TMB), microsatellite instability (MSI) scores, and corresponding clinical information for 33 cancer types, including bladder urothelial carcinoma (BLCA), were systematically retrieved from the University of California Santa Cruz (UCSC) Xena platform (https://xenabrowser.net/datapages/) [7,8]. Specifically, we utilized the “TCGA TARGET GTEx” cohort for pan-cancer expression analysis. Only samples with complete clinical annotation were included in the survival and correlation analyses. For independent validation of expression patterns and prognostic evaluation of ICAM5, we additionally downloaded level 3 RNA-Seq data (HTSeq-FPKM) and clinical metadata for bladder cancer and other selected malignancies from The Cancer Genome Atlas (TCGA) via the Genomic Data Commons (GDC) portal (https://portal.gdc.cancer.gov/). All expression data were log2-transformed (log2[FPKM+1]) to approximate a normal distribution for downstream statistical analysis. Samples labeled as “Solid Tissue Normal” were classified as controls, while primary tumor samples were used for tumor group analyses. Any batch effects across different datasets were assessed and are noted in the respective analysis sections.

### Differential Expression Analysis and Validation of ICAM5

A p value < 0.05 and an absolute fold change > 1.5 were applied to assess the differences in the expression levels of ICAM5 across cancers in the TCGA database. We used the “limma” package in R 4.1.1 (http://www.r-project.org/) to obtain the pancancer expression value for ICAM5. We utilized the Wilcoxon rank sum test to assess ICAM5 expression levels in tumor and normal tissues. The comparisons were visualized via the R package ggplot2, while statistical significance was defined as a p value < 0.05. The mRNA expression specificity of ICAM5 in various clinical samples from BCa patients was further investigated.

### Survival Prognostic Analysis

Survival statistics derived from the samples in the TCGA database. The correlations between ICAM5 expression and overall survival (OS), disease-specific survival (DSS), the progression-free interval (PFI), and the disease-free interval (DFI) were assessed via Cox proportional hazards model analysis. Kaplan‒Meier (KM) curve analysis was used to assess the connection between the ICAM5 transcript level and tumor prognosis. Statistical analysis was conducted via the “survival” package, and the data were visualized via the “survminer” package.

### Weighted gene coexpression network analysis (WGCNA) and enrichment pathway analysis

Weighted gene co-expression network analysis (WGCNA) is a systems biology tool that identifies gene sets with synergistic changes by analyzing gene relationship patterns across samples. WGCNA was performed using the WGCNA R package (v1.72-5). Differentially expressed protein-coding genes with expression variance in the top 50% were selected for network construction. A soft-threshold power (β) of 3 was applied to achieve scale-free topology (fit index > 0.85). From this, an unsigned adjacency and topological overlap matrix (TOM) were created. Gene modules were identified via hierarchical clustering of TOM dissimilarity (minimum module size = 30; merge cut height = 0.25). Module eigengenes were correlated with key clinical traits using Pearson correlation. The most clinically relevant module (blue) was selected for downstream analysis.. Functional enrichment of the key module genes was conducted using the Gene Set Cancer Analysis (GSCA) platform. In the “Expression” module, the “Expression & Pathway Activity” function was utilized to analyze input gene names. This analysis determines the percentage of selected cancer types in which the mRNA expression of each gene is significantly associated with pathway activity. Specifically, a gene is included in the figure only if it demonstrates a significant association (FDR ≤ 0.05) with at least one pathway in at least one cancer type; the numeric value within each cell represents this percentage across the evaluated cancer types. Concurrently, Gene Set Enrichment Analysis (GSEA) was performed using the Hallmark (v2023.1) and KEGG pathway gene sets. Statistical significance was assessed using a hypergeometric test with false discovery rate (FDR) correction; pathways with an FDR < 0.05 were defined as significant and reported.

### Cell Cultures

Two cell lines for bladder cancer (namely, 5637 and T24) were obtained from Procell Life Science & Technology Co., Ltd. (Wuhan, China). In Roswell Park Memorial Institute (RPMI)-1640 media (Gibco, USA) supplemented with 10% fetal bovine serum (BioInd, Israel), the cell lines were cultivated at 37 °C with 5% CO2 in the environment.

### RNA Extraction and Real-time Polymerase Chain Reaction (PCR)

Cellular RNA was extracted via TRIzol reagent. The sequences of primers used for real-time quantitative polymerase chain reaction were obtained from Applied Biosystems as follows: ICAM5 (forward-5’-AGAACAGGAAGGCACCAAACAG-3’; reverse-5’- CTGGCTCACTCAAAGTCAGAAGAG-3’) and glyceraldehyde 3-phosphate dehydrogenase (GAPDH) (forward-5’-GGAGCGAGATCCCTCCAAAAT-3’; reverse-5’-GGCTGTTGTCATACTTCTCATGG-3’). The change in gene expression was quantified via normalization to the expression of GAPDH via the 2^-∆∆Ct^ method.

### Cell Counting Kit-8 (CCK-8) Assay

At a density of 3 × 10^3^ cells/well, two transfected bladder cancer cell lines were moved to 96-well plates. After 0, 1, 2, 3, and 4 days, the viability of the cells was evaluated. To each well, CCK-8 solution (10 μL) was added. An incubator for cells was used to hold the 96-well plates. After 60 minutes, the absorbance was measured at 450/570 nm.

### Wound Healing Assay

Six-well plates were seeded with transfected 5637 and T24 cells. Upon reaching confluence, a 200-μL pipette tip was used to form three lines of scratches in each well. The cells were incubated with 1% FBS. The samples were observed with a microscope (IX51, Olympus, Japan) for 0 and 24 hours.

### 5-Ethynyl-2’-Deoxyuridine (EdU) Assays

Cell proliferation was also evaluated using EdU assays, following the instructions of the manufacturer regarding the use of the Cell Light EdU Apollo567 In Vitro Imaging Kit. Briefly, 96-well plates were planted with 3 × 10^3 transfected cells per well of the 5637 and T24 cells. The cells were immediately treated with EdU for two hours, fixed with 4% paraformaldehyde, and stained with the kit, as directed by the manufacturer. An Olympus microscope was used to capture images.

### Cell Migration and Invasion Assay

Cell migration and invasion were measured in 24-well Transwell chambers with 8 μm pores. The transfected T24 and 5637 cells were inserted into the upper chamber (without or with Matrigel gel) at a density of 2 × 10^4^ cells/200 μL. Then, RPMI medium infused with 10% FBS was added to the lower chamber. After being incubated at 37 °C for 24 hours, the cells were fixed in the lower chamber with 4% paraformaldehyde, dyed with 0.1% crystal violet, and counted.

### Differences in the tumor microenvironment and immunotherapy

The relationship between ICAM5 expression and immune infiltration was evaluated using three computational methods.First, stromal and immune scores were estimated with the ESTIMATE algorithm via the estimate R package (v1.0.13) based on TPM-normalized expression data. Second, immune cell subtype abundance was quantified using single-sample Gene Set Enrichment Analysis (ssGSEA) implemented with the GSVA R package (v1.46.0). Finally, correlations between ICAM5 expression and infiltration levels of major immune cell types were validated using the TIMER2.0 web tool (http://timer.cistrome.org), which provides data on immune cell infiltration in various cancer types, the association between the degree of immune cells and ICAM5 expression was computed [9]. In addition, the correlations between ICAM5 and tumor neoantigen burden (TNB), tumor mutation burden (TMB), microsatellite instability (MSI), treatment response to immune checkpoint inhibitors (ICIs) and related immune checkpoint genes (including PDCD1, IL10, CD274, CTLA4, LAG3, TGFB1, CD276, VEGFB, IDO1 and HAVCR2) were evaluated. To validate the association between ICAM5 expression and immunotherapy response, we analyzed an independent public cohort of advanced urothelial carcinoma patients treated with anti-PD-1/PD-L1 inhibitors. Gene expression data and clinical response annotations were derived from the TCGA-BLCA cohort. Raw RNA-seq counts from pre-treatment samples were normalized using the DESeq2 median-of-ratios method. Patients were classified as complete responders (CR) or partial responders (PR) based on RECIST v1.1 criteria for comparative analysis. Samples were further stratified into immune-inflamed, immune-excluded, and immune-desert phenotypes using established transcriptomic signatures to evaluate ICAM5 expression across distinct tumor immune microenvironments.

### Comparison of ICAM5 expression and methylation

Data from the TCGA database were evaluated to determine the link between TRIP13 and RNA-modified genes across cancers. The expression levels of M1A-related genes (including TRMT6 and ALKBH1), m5C (including NSUN2 and ALYREF), and m6A (including WTAP, METTL14 and IGF2 BP1) and ICAM5 were further analyzed.

### Genetic Mutation Analysis

We obtained all TCGA samples processed with MuTect2 software [10] from Genomic Data Commons (GDC) (https://portal.gdc.cancer.gov/) and integrated the mutation data from the samples. The variation in the rate of gene mutations in each sample group was assessed using a chi-square test. A total of 405 samples with mutations were found, of which 318 (78.5%) were plotted. We also investigated the associations between the ICAM5 expression level and different mutation types, including copy number variations (CNVs) and single-nucleotide variations (SNVs). After downloading the information from the UCSC database, we retrieved the ICAM5 expression information from the different samples. Moreover, using R software, we computed the variations in ICAM5 expression across various clinical stage BCa samples by combining the mutation and gene expression data of the samples, filtering the samples with synonymous mutations. The significance of differences between pairs was examined using the unpaired Wilcoxon rank sum and signed rank tests, while differences between several sample groups were evaluated using the Kruskal test.

### Statistical Analysis

Differences in ICAM5 expression across groups were analyzed using one-way ANOVA, while categorical data were compared using the Chi-square test. Univariate and multivariate Cox proportional hazards models were applied to calculate hazard ratios (HRs) with 95% confidence intervals, and effect sizes were reported for key comparisons. Statistical significance was defined as a two-sided *p*‑value < 0.05. The false discovery rate (FDR) method (Benjamini–Hochberg procedure) was applied to correct for multiple testing in correlation analyses and functional enrichment results; uncorrected *p*‑values are reported only for pre‑specified primary analyses, such as survival comparisons. Image quantification was performed using ImageJ (v2.3.0). All statistical analyses were conducted in R (v4.2.1). all in vitro assays were performed with three independent biological replicates. Data were presented as the mean ± standard deviation. Statistical comparisons between groups were analyzed using a two-tailed Student’s t-test with GraphPad Prism 9.0 software, and a p-value < 0.05 was considered statistically significant.

## 3. Results

### ICAM5 is highly expressed in bladder cancer and is correlated with clinical features

**Fig 1:**
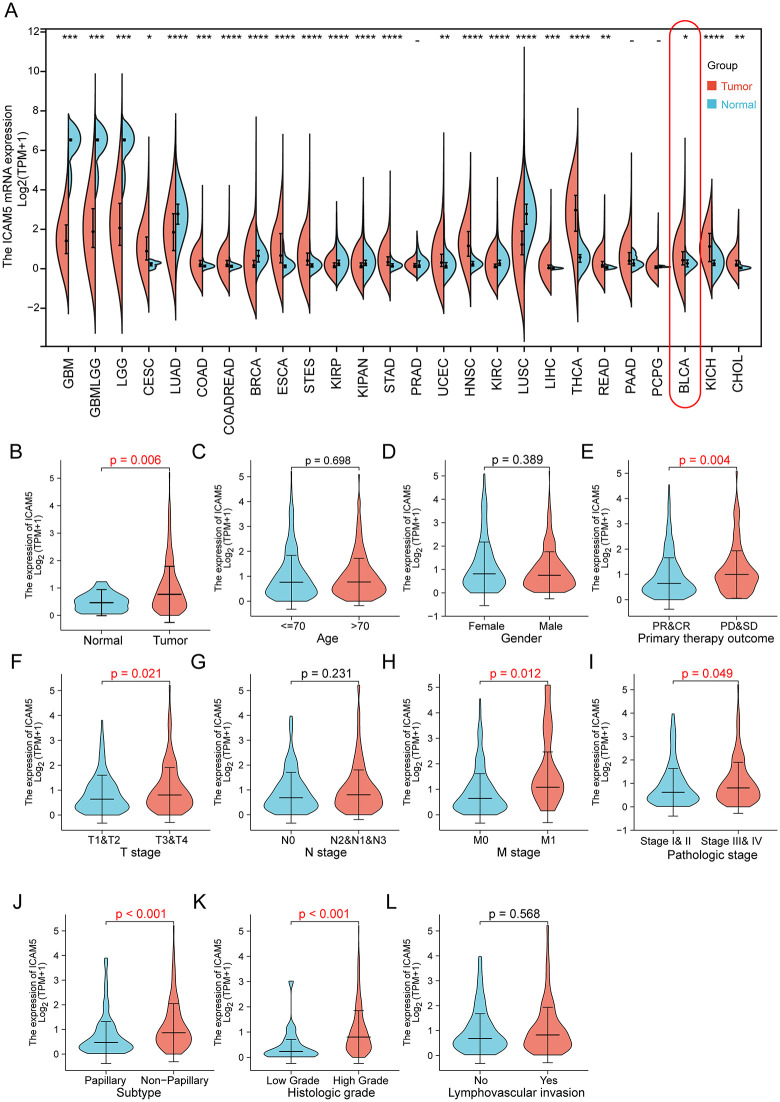
ICAM5 is aberrantly expressed in bladder cancer and is correlated with clinical characteristics. **(A)** ICAM5 expression levels across cancers. **(B)** ICAM5 expression levels in bladder cancer tissues and paired normal tissues. **(C-L)** Relationships between the ICAM5 expression level and age, sex, primary therapy outcome, TNM stage, pathologic stage, subtype, histologic grade and lymphovascular invasion in patients with BCa.

The UCSC Xena database provided transcriptome data for 26 different forms of cancer. First, we utilized the online databases of the TCGA and GEtX projects to investigate the expression patterns of ICAM5 across tumor and normal samples via pancancer analysis. Our analysis revealed an upregulation of ICAM5 in tumor tissues, particularly in bladder cancer samples (**Fig 1A**). Interestingly, a significant increase in ICAM5 expression was specifically observed in bladder cancer tissues compared with matched normal tissues (**Fig 1B**). Supplementary table 1 summarizes the clinical characteristics of bladder cancer patients stratified into ICAM5-high and ICAM5-low subgroups. The findings revealed that there were substantial differences in ICAM5 expression levels across bladder cancer subtypes, stages, pathologic stages, primary therapeutic outcomes, and histologic grades. These results indicate that ICAM5 might be a crucial player for the pathogenesis and development of BCa (Figs 1C-L).

### ICAM5 is associated with the prognosis of several tumors

**Fig 2:**
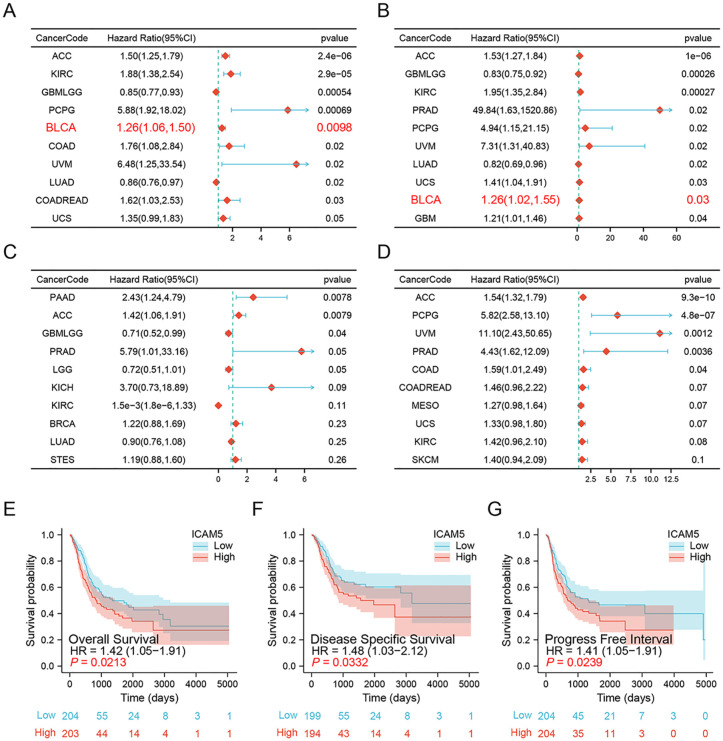
Prognostic Value of ICAM in BCa. **(A-D)** Forest maps showing OS, DSS, PFI and DFI for ICAM5 across cancers. **(E-G)** Kaplan–Meier analysis of the associations between ICAM5 expression and OS, DSS and PFI in patients with BCa.

We further assessed the prognostic value of ICAM5 across cancers in terms of overall survival (OS), disease-specific survival (DSS), disease-free interval (DFI), and progression-free interval (PFI). We identified ICAM5 as an increased risk indicator of OS in ten forms of cancer, DSS in ten forms of cancer, DFI in five forms of cancer, and PFI in five forms of cancer (Figs 2A-D). Notably, ICAM5 was identified as a risk factor for both OS and DSS in bladder cancer patients. According to Kaplan-Meier analysis, patients with bladder cancer who had higher ICAM5 expression had lower OS, DSS, and PFI (Figs 2E-G). As shown in supplementary table 2, clinical factors, such as M stage, primary therapy outcome, histologic grade, and tumor subtype, were significantly correlated with ICAM5 expression levels (p < 0.05) according to single gene logistic model analysis. Univariate Cox regression correlation analysis revealed that clinical factors, including TM stage, clinical stage, tumor subtype and the expression of ICAM5, were significantly related to OS. Subsequent multivariate Cox regression analysis revealed that only ICAM5 expression was notably associated with OS. It was discovered that elevated ICAM5 expression constituted a separate risk factor for poor OS through supplementary table 3.

### ICAM5 activates the EMT process in bladder cancer

**Fig 3:**
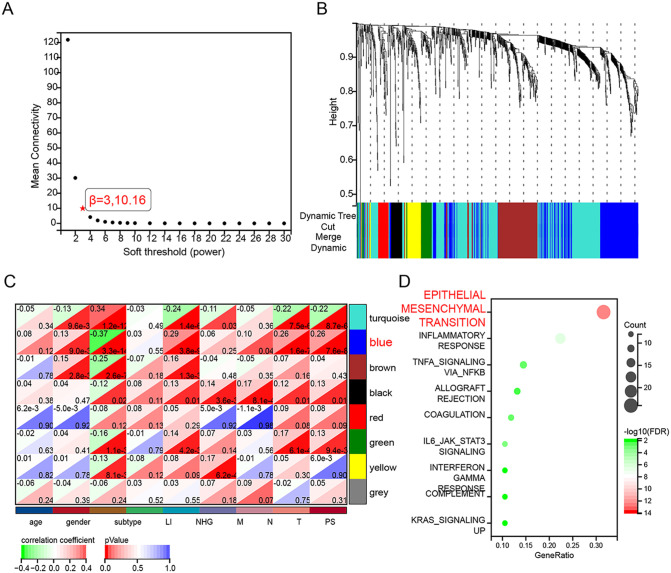
Soft threshold calculation and screening of key modules in weighted gene coexpression network analysis (WGCNA)(A) Mean connectivity of each soft threshold in BCa. **(B)** Gene cluster dendrogram generated by hierarchical clustering based on the dissimilarity measure. Eight modules are assigned different colors. **(C)** Heatmap of module-trait relationships. The darker the module color is, the stronger the correlation between the two. **(D)** Bubble map of the 9 ICAM5-related signaling pathways identified via KEGG analysis.

To establish a weighted gene coexpression network, we used differentially expressed genes (DEGs) derived from the TCGA Bladder Cancer expression matrix. The gene coexpression network was created using a soft threshold of three (**Fig 3A**), ensuring the inclusion of genes with similar expression patterns within the same module. We subsequently identified a total of eight gene modules through the analysis (**Fig 3B**), with each module demonstrating associations with various clinical features, such as age, sex, subtype, lymphovascular invasion (LI), and neoplasm histologic grade (NHG), as well as pathologic M (M), pathologic N (N), pathologic T (T), and pathologic stage (PS) (**Fig 3C**). Notably, the blue module showed a deep color in relation to clinical characteristics, indicating its importance for further analyses. This module was linked primarily to nine signaling pathways, with a predominant focus on the EMT process (**Fig 3D**).

### ICAM5 regulates different pathways in cancers

**Fig 4:**
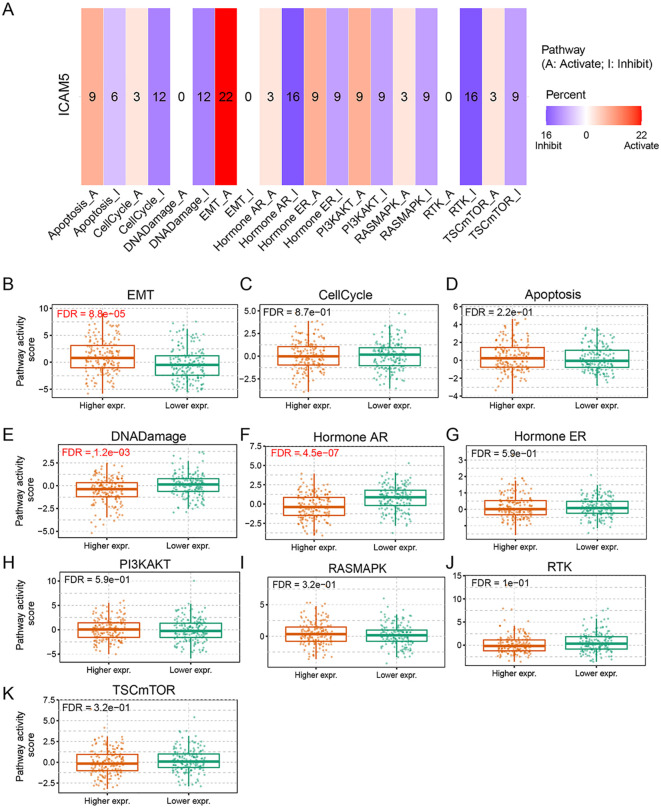
ICAM5-related signaling pathways in cancers. **(A)** Different signaling pathways related to ICAM5 in 32 tumors. **(B)** ICAM5-related signaling pathways in BCa, such as the EMT, cell cycle, apoptosis, DNA damage, hormone AR, hormone ER, PI3KAKT, RASMAPK, RTK, and TSC mTOR pathways.

To investigate the impact of ICAM5 on various signaling pathways in 32 different tumors, we employed gene set cancer analysis (GSCA) to elucidate the role of ICAM5. Our findings revealed that ICAM5 acts as an inhibitor of DNA damage and the receptor tyrosine kinase (RTK) signaling pathways, whereas it promotes the EMT process (**Fig 4A**). The impacts of ICAM5 on numerous signaling processes, including EMT, the cell cycle, apoptosis, DNA damage, hormone AR, hormone ER, PI3K-AKT, RAS-MAPK, RTK, and TSC-mTOR, were then thoroughly investigated, with a focus on bladder cancer (Figs 4B-K). Our results indicated that ICAM5 acts as an activator of the EMT pathway (**Fig 4B**) but as an inhibitor of the DNA damage (**Fig 4E**) and hormone AR (**Fig 4F**) pathways in bladder cancer. The observed results imply that ICAM5 might be important for the DNA damage and EMT procedures occurring in bladder cancer. Interestingly, ICAM5 had no significant effect on the RTK pathway (**Fig 4J**), highlighting its specific roles in different signaling cascades in bladder cancer.

### ICAM5 regulates bladder cancer proliferation, invasion and migration

**Fig 5:**
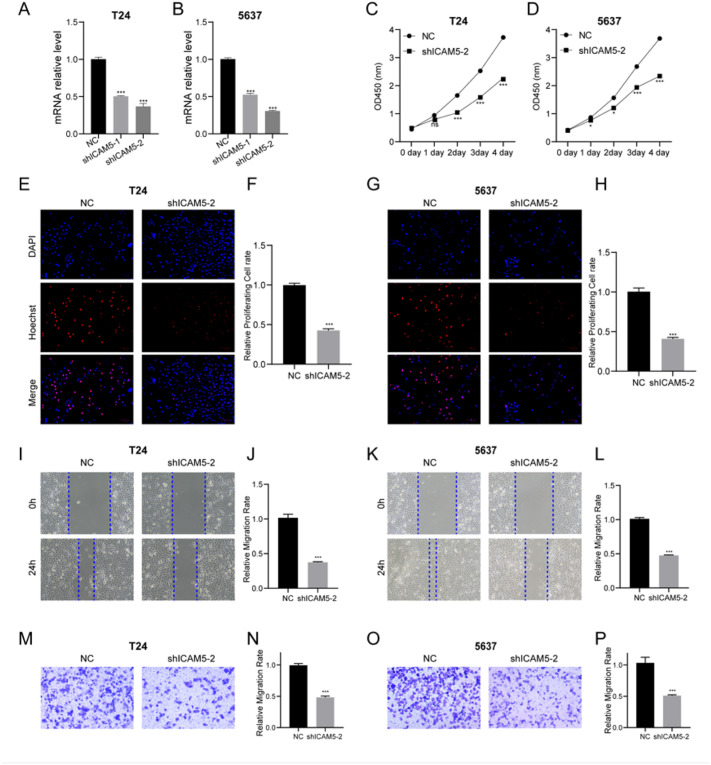
The function of ICAM5 in bladder cancer cell lines. **(A, B)** ICIAM5 expression was knocked down by sh-ICIAM5 in both the T-24 and 5637 cell lines. **(C, D)** Proliferation of sh-ICIAM5-transfected T-24 and 5637 cells, as evaluated by a Cell Counting Kit-8. **(E-H)** Proliferation of sh-ICIAM5-transfected T-24 and 5637 cells, as evaluated by EDU. **(I-L)** Migration of sh-ICIAM5-treated T-24 and 5637 cells, as assessed by wound healing. **(M-P)** Invasion of sh-ICIAM5-treated T-24 and 5637 cells, as assessed by Transwell experiments.

To downregulate ICAM5 expression, shRNA plasmids targeting ICAM5 (sh-ICAM5) were transfected into both T-24 and 5637 cell lines (Figs 5A and 5B). The resulting knockdown was validated at the protein level by western blot analysis in 5637 cells (S1 Fig), and the reduction in ICAM5 protein was consistent with the decrease observed at the mRNA level. The viability of both the T-24 and 5637 cells was considerably decreased in the sh-ICAM5 groups compared to the NC groups (Fig 5C, 5D). EdU analysis revealed that the proliferation of BCa cells was significantly decreased when ICAM5 was knocked down (Figs 5E-5H). Furthermore, we assessed the impacts on invasion and motility after ICAM5 knockdown in BCa cells. Our results indicated that cell migration in the sh-ICAM5 group was substantially lower than that in the control group (Figs 5I-5L), and a similar change in the invasion rate was also observed (Figs 5M-5P).

### ICAM5 correlates with specific immune cell infiltration patterns

**Fig 6:**
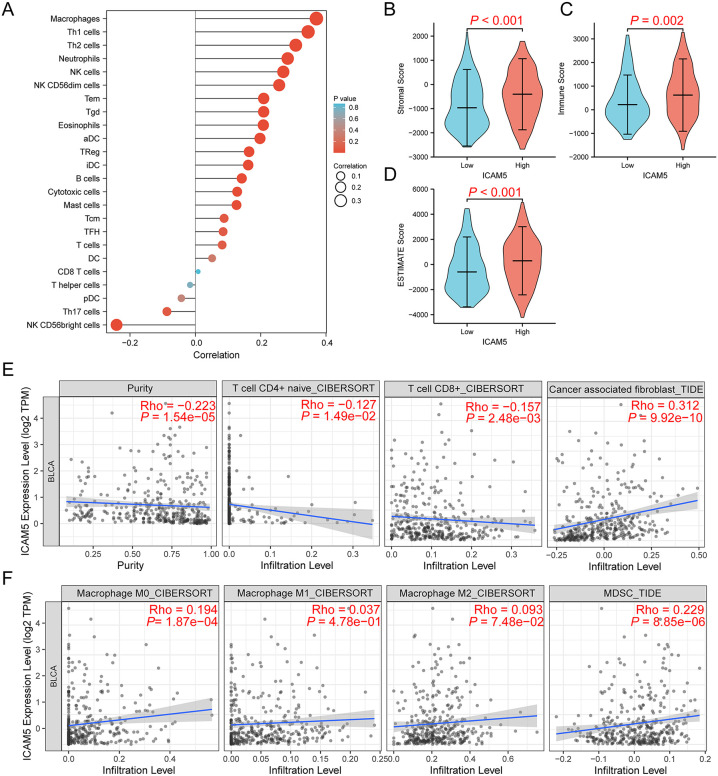
Immune characteristics of ICAM5 in bladder cancer. (**A**) ssGSEA. **(B-D)** StromalScore, immuneScore, and ESTIMATE score of BCa. **(E-F)** Correlations between ICAM5 expression and immunocyte infiltration, such as purity, CD4 + T cells, CD8 + T cells, cancer-associated fibroblasts, M0-2 macrophages and MDSCs, in BCa.

We further explored the role of the immune microenvironment in bladder cancer and its relationship with ICAM5 expression. Using the single-sample gene set enrichment analysis (ssGSEA) algorithm, we calculated immunocyte signatures and analyzed their associations with ICAM5 expression in bladder cancer. The results revealed a positive correlation between ICAM5 and 19 types of immunocytes, as well as a negative correlation with 2 types of immunocytes in bladder cancer (**Fig 6A**). We subsequently investigated the impact of ICAM5 expression on overall immune characteristics and immune cell infiltration in bladder cancer samples with varying risk scores. Comparing the high-expression group with the low-expression group, we observed elevated stromal scores (p < 0.001), immune scores (p = 0.002), and ESTIMATES scores (p < 0.001) in the high-expression one (Figs 6B-D). These findings may suggest that increased ICAM5 expression may be associated with a more pronounced immune microenvironment in bladder cancer. The TIMER 2.0 database was utilized to assess the potential relationship between ICAM5 expression and immunocyte infiltration. Our analysis revealed ICAM5 expression was found to be inversely correlated with the infiltration of CD4 + naive T cells and CD8 + T cells (p < 0.001, **Fig 6E**), whereas a positive association was observed with cancer-related fibroblasts, M0 macrophages, and myeloid-derived suppressor cells (MDSCs) (p < 0.05, **Fig 6F**). These results might provide insights into the complex interplay between ICAM5 expression and immune cell infiltration in the context of bladder cancer.

### Association of ICAM5 with immune checkpoints and therapeutic response

**Fig 7:**
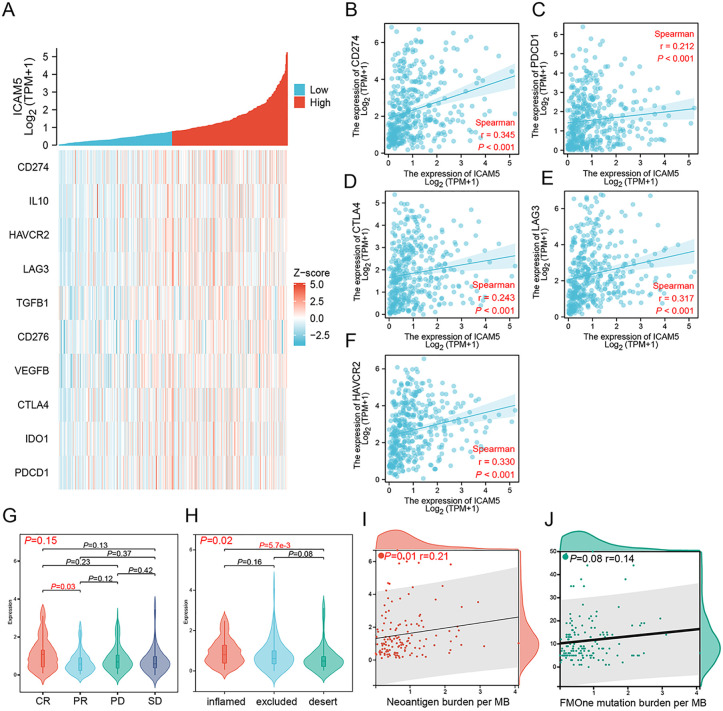
Correlations between ICAM5 and BCa immune checkpoints and immunotherapy in bladder cancer. **(A)** Coexpression heatmap of ICAM5 and immune checkpoints. **(B-F)** Scatterplots of the correlations between ICAM5 and immune checkpoints. **(G)** Correlations of ICAM5 with complete response (CR), partial response (PR), stable disease (SD) and progressive disease (PD) with anti-PD-L1 immunotherapy. **(H)** Correlations between ICAM5 and three immune response phenotypes, namely, inflamed, excluded and desert. **(I)** Correlation between ICAM5 and TNB. **(J)** Correlation between ICAM5 and TMB.

We examined at the relationships of ICAM5 and immunological checkpoints in bladder cancer, focusing on their potential implications for immunotherapy. Results showed ICAM5 levels were found to be strongly associated with raised expression of immunological checkpoints in bladder cancer, including PDCD1 (PD-1), CD274 (PD-L1), HAVCR2, LAG3, and CTLA4 (Figs 7A-7F). The results presented imply that ICAM5 may be involved in modulating the expression of crucial immune checkpoint molecules within the tumor microenvironment. Furthermore, through gene expression profiling in an anti-PD-L1 treatment cohort of bladder cancer patients, we observed that patients with a complete response to immunotherapy expressed higher levels of ICAM5 than those with a partial response (**Fig 7G**). Additionally, the geometric mean expression value of ICAM5 was significantly greater in the immune-inflamed phenotypes than in the immune-excluded and immune-desert phenotypes (**Fig 7H**). Additionally, we found that ICAM5 expression and tumor neoantigen burden (TNB) were positively associated in patients with bladder cancer (**Fig 7I**). Moreover, we assessed the relationship between ICAM5 and tumor mutation burden (TMB) in bladder cancer. Our analysis, which utilized the TMB package in R, revealed a close association between ICAM5 expression and TMB in bladder cancer (**Fig 7J**). These results suggest that ICAM5 may serve as a potential biomarker for predicting the response to immunotherapy and could be involved in immune modulation within the tumor microenvironment of bladder cancer.

### The relationship between ICAM5 expression and methylation regulation

**Fig 8:**
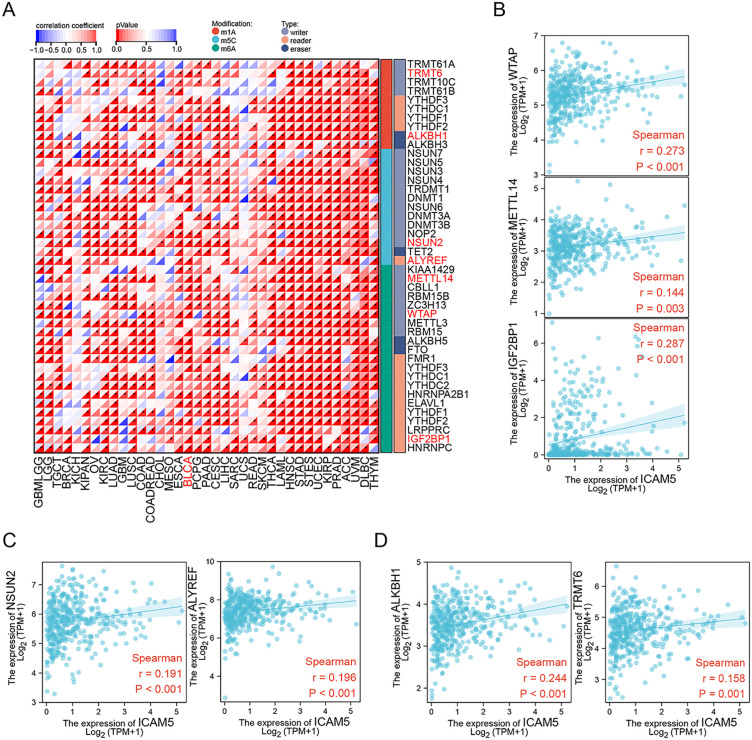
Association of ICAM5 with RNA modifications. **(A)** Association of ICAM5 with RNA modifications across cancers. **(B-D)** Association of ICAM5 with m6A, m5C, and m1A-related genes in BCa.

To investigate the potential regulatory mechanisms that influence ICAM5 expression, we investigated the correlations among DNA methylation levels, RNA modifications, and ICAM5 expression. Analysis across cancer types showed coordinated expression of ICAM5 with key RNA modification genes (m1A, m5C, m6A) (Fig 8A). This consistent co-expression pattern merits further investigation to elucidate any potential functional interplay between these pathways. Furthermore, in bladder cancer specifically, we identified DNA methylation-related genes, such as WTAP, METTL14, IGF2 BP1, NSUN2, ALYREF, ALKBH1, and TRMT6, that exhibited a significant positive correlation with ICAM5 expression (Figs 8B-D). These findings suggest that in the context of bladder cancer, ICAM5 expression may be regulated primarily by RNA posttranscriptional modifications. This comprehensive analysis elucidates the potential regulatory mechanisms that influence ICAM5 expression levels to provide insights into the molecular pathways involved in bladder cancer progression.

### The mutational landscape of ICAM5 in bladder cancer

**Fig 9:**
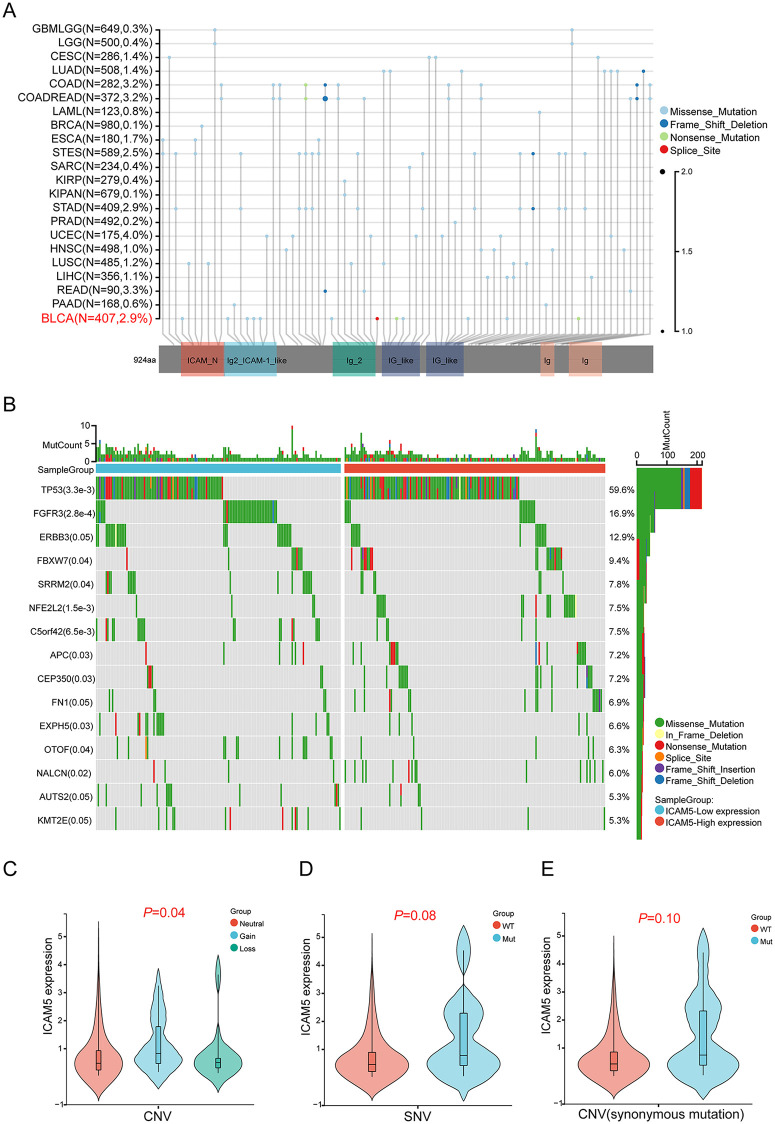
Mutation characteristics of ICAM5 in BCa. **(A)** The alteration frequency with mutation type across cancers. **(B)** Mutated genes of the ICAM5-low and high groups in BCa. **(C)** Association between ICAM5 expression levels and the neutral, gain, and loss in CNV groups. **(D-E)**. Associations of the wild-type and mutant groups with the expression level of ICAM5 with synonymous mutations and CNV events.

We evaluated the types of mutations, the frequency of gene alteration, and the posttranslational modification (PTM) sites using information from the Genomic Data Commons (GDC) in order to analyze the genetic alteration status of ICAM5 in bladder cancer. The outcomes revealed gene alterations in ICAM5 across various cancers, including missense mutations, frameshift deletions, nonsense mutations, and splice site mutations. The highest gene alteration frequencies for ICAM5 were noted in COAD (3.2%), COADREAD (3.2%), and bladder cancer (BCa) (2.9%) (Fig 9A). Furthermore, we identified several significantly mutated genes in both the high- and low-ICAM5 expression groups (Fig 9B), with missense mutations being predominant in bladder cancer patients compared with other types of genetic alterations. Notably, the potential implications of FGFR3 and NALCN in bladder cancer pathology are highlighted. Additionally, we explored the associations between ICAM5 expression levels and different mutation types. Our analysis revealed a positive correlation between ICAM5 expression levels and copy number variation (CNV) as well as single-nucleotide variation (SNV) percentages in bladder cancer. Specifically, higher ICAM5 expression levels were associated with more frequent CNV events in bladder cancer, suggesting a potential regulatory relationship (Figs 9C and 9D). However, ICAM5 expression levels did not significantly differ between the wild-type and mutant groups with respect to frequent synonymous mutations and CNV events in bladder cancer (Figs 9D and 9E).

## 4. Discussion

Bladder cancer is the second most common urological malignancy globally, accounting for 549,000 new cases and approximately 200,000 deaths every year [11]. The degree of tumor invasion can be used to distinguish between two types of BCa: muscle-invasive bladder cancer (MIBC) and nonmuscle-invasive bladder cancer (NMIBC). Patients with NMIBC frequently experience recurrence or progression, with five-year rates ranging from 31–78% and 1–45%, respectively [12]. This clinical challenge is particularly evident among patients with high HER2 expression [13,14]. Patients’ financial burden is exacerbated by the high rates of BCa development and recurrence since they require multiple tests and treatments [15].

Intercellular adhesion molecules (ICAMs) are known to be involved in various human cancers [16,17]. ICAM5, a member of the ICAM family of adhesion proteins, is expressed mainly in specific areas of the brain [18]. One study revealed that ICAM5, which is associated with a significantly increased methylation rate, might play a role in the high incidence and aggressiveness of colorectal cancer in the AA population [19]. Another study indicated that a variant in ICAM5 was significantly associated with breast and prostate cancer risk and affected disease progression and prognosis [20].

The current research involved a thorough bioinformatics analysis of ICAM5 in various cancers, including tumor immune infiltration cells, the response to ICIs, gene expression analysis in various tissues and cell lines, gene mutation, DNA methylation, prognostic value and its association with clinical features. Our results revealed elevated levels of ICAM5 in multiple tumor tissues, including bladder cancer. Moreover, the ICAM5 expression level is associated with diverse clinical characteristics of BCa, such as different stages, pathologic stages, subtypes, and histologic grades. Furthermore, high ICAM5 expression was negatively associated with OS, DSS, and the PFI in patients with bladder cancer when its prognostic value was explored. Thus, analogous to the established marker HER2, ICAM5 shows significant prognostic potential in bladder cancer, warranting further investigation.

To specifically elucidate the functional implications of ICAM5 overexpression in BCa, we transitioned from the pan-cancer overview to a detailed investigation of its potential oncogenic mechanisms. Previous studies have demonstrated that ICAM5 contributes to tumor progression through distinct signaling pathways depending on the cancer type. For example, in thyroid carcinoma, ICAM5 activates the MAPK/ERK and MAPK/JNK pathways [21], While, in bladder cancer, ICAM5, acting as a downstream effector of the AKT/mTOR signaling pathway, can influence the tumor progression [22]. Additionally, ICAM5 has been implicated in colorectal cancer progression by promoting epithelial-mesenchymal transition (EMT) and enhancing stemness [23]. In line with these findings and informed by our pathway enrichment analysis, our results suggest that in BCa, ICAM5 expression is associated with the regulation of the DNA damage and RTK signaling pathways and may contribute to the activation of the epithelial‒mesenchymal transition (EMT) process. Additionally, cellular experiments demonstrated that ICAM5 promoted bladder cancer cell proliferation, migration and invasion. This functional validation aligns with the pro-tumorigenic role for ICAM5 in BCa. However, However, the precise mechanisms and pathways through which ICAM5 functions warrant further investigation, including future studies employing independent shRNAs or rescue experiments to solidify the causal relationship.

Given the established link between EMT, tumor progression, and immune modulation, we next investigated the relationship between ICAM5 and the tumor immune microenvironment (TME) in BCa. The immune TME, which is primarily characterized by tumor-infiltrating immune cells (TIICs), is critical for cancer therapy and prognosis [24,25]. Increased levels of TIICs within the TME are associated with better outcomes in several types of cancer [26,27]. Multiple immunosuppressive cell populations—such as cancer-associated fibroblasts (CAFs), tumor-associated macrophages (TAMs), and myeloid-derived suppressor cells (MDSCs)—act as key drivers of tumor immune evasion [28–30].Tian et al. reported that the soluble form of ICAM-5 attenuates the T-cell receptor-mediated activation of T cells, as evidenced by decreased expression of activation markers. This finding suggested that ICAM-5 is involved in the immune function of the brain [31]. Paetau et al. also reported that ICAM-5 can decrease the release of the proinflammatory cytokines tumor necrosis factor α (TNF-α) and interleukin 1β (IL-1β) but can induce the release of the anti-inflammatory cytokine IL-10 when the brain is under immune challenge [32]. However, studies on the relationship between ICAM5 and immune cell infiltration in tumors are scarce. Aligned with prior findings regarding ICAM5 in LUAD [33,34], our study extends this knowledge to BCa, revealing that ICAM5 expression is significantly correlated with immune cell infiltration, including that of CD4 + naive T cells, CD8 + T cells, CAFs, macrophages, and MDSCs. Importantly, the pattern of association—showing strong positive correlations with immunosuppressive cell types (CAFs, MDSCs) and key inhibitory checkpoints (PD-1, PD-L1, CTLA4, LAG3, HAVCR2)—suggests that ICAM5 may be associated with an immunosuppressive TME in bladder cancer, potentially contributing to immune evasion. This finding may explain how ICAM5 influences immunological responses. Thus, ICAM5 appears to be a viable therapeutic target for cancer.

Building on the connections between ICAM5, immune evasion, and tumor progression, we further explored potential genomic and epigenomic features in BCa. Epigenetic alterations influence tumor progression by influencing gene function and expression levels, mostly through DNA methylation, histone modification, noncoding RNA control, and chromatin structure remodeling. A previous study revealed that the transcriptional activation of ICAM5 and promoter hypermethylation are mediated by DNMT1 and DNMT3a in THCA, which facilitate the progression of cancer [21]. Our analysis revealed notable genetic and epigenetic features associated with ICAM5 in BCa, specifically showing positive correlations with most RNA modification-related genes as well as with CNV and SNV percentages. Moreover, BCa had the highest incidence of ICAM5 gene mutations among the cancers analyzed. These observations lead us to speculate that ICAM5 may be involved in or associated with the landscape of gene mutations and epigenetic changes in bladder cancer. However, additional evidence is needed to confirm the potential role of ICAM5 DNA methylation and genetic alteration status in the tumorigenesis of BCa.

In summary, our integrated multiomics and functional study elucidates the tumor-specific significance of ICAM5 in bladder cancer. We demonstrate that elevated ICAM5 expression serves as a marker of poor prognosis and is correlated with the activation of pro-tumorigenic pathways, an immunosuppressive tumor microenvironment, and distinct genetic/epigenetic features. These converging lines of evidence position ICAM5 as a multifunctional hub associated with bladder cancer progression. Consequently, ICAM5 emerges as a compelling candidate biomarker and a rational therapeutic target warranting further mechanistic and clinical investigation.

## Supporting information

S1 Table The clinical characteristics of ICAM5 expression in bladder cancer patients. (DOCX)

S2 Table The relationship between ICAM5 and clinical factors via single gene logistic model analysis in bladder cancer. (DOCX)

S3 Table Cox regression model analysis of overall survival in patient with bladder cancer. (DOCX)

S1 Fig Protein-Level Validation of ICAM5 Knockdown in 5637 Cells by Western Blot. (DOCX)

S1 Data The raw data of Western Blot. (DOCX)
