# Supplementary material for: Elevated ICAM5 as a promising predictor of poor prognosis in bladder cancer via EMT, immune microenvironment, and therapy resistance
Source: PLoS One. 2026 Jun 8;21(6):e0347623. doi: 10.1371/journal.pone.0347623 (PMC13245789; doi:10.1371/journal.pone.0347623)
Supplement: S1 Table — (DOCX) [file pone.0347623.s001.docx]

Supplementary table 1. The clinical characteristics of ICAM5 expression in bladder cancer patients

| Characteristic | Low expression | High expression | *P* |
| --- | --- | --- | --- |
| n | 204 | 204 |  |
| Gender, n (%) |  |  | 0.653 |
| Female | 51 (12.5%) | 56 (13.7%) |  |
| Male | 153 (37.5%) | 148 (36.3%) |  |
| T stage, n (%) |  |  | 0.497 |
| T1 | 1 (0.3%) | 2 (0.5%) |  |
| T2 | 66 (17.6%) | 53 (14.2%) |  |
| T3 | 91 (24.3%) | 103 (27.5%) |  |
| T4 | 28 (7.5%) | 30 (8%) |  |
| N stage, n (%) |  |  | 0.146 |
| N0 | 124 (33.9%) | 113 (30.9%) |  |
| N1 | 21 (5.7%) | 25 (6.8%) |  |
| N2 | 39 (10.7%) | 36 (9.8%) |  |
| N3 | 1 (0.3%) | 7 (1.9%) |  |
| M stage, n (%) |  |  | **0.006** |
| M0 | 111 (53.6%) | 85 (41.1%) |  |
| M1 | 1 (0.5%) | 10 (4.8%) |  |
| Pathologic stage, n (%) |  |  | 0.316 |
| Stage I | 1 (0.2%) | 1 (0.2%) |  |
| Stage II | 73 (18%) | 57 (14%) |  |
| Stage III | 66 (16.3%) | 74 (18.2%) |  |
| Stage IV | 62 (15.3%) | 72 (17.7%) |  |
| Histologic grade, n (%) |  |  | **0.006** |
| High Grade | 184 (45.4%) | 200 (49.4%) |  |
| Low Grade | 17 (4.2%) | 4 (1%) |  |
| Subtype, n (%) |  |  | **< 0.001** |
| Non-Papillary | 117 (29%) | 154 (38.2%) |  |
| Papillary | 86 (21.3%) | 46 (11.4%) |  |
| Lymphovascular invasion, n (%) |  |  | 0.372 |
| No | 69 (24.6%) | 61 (21.7%) |  |
| Yes | 71 (25.3%) | 80 (28.5%) |  |
| Primary therapy outcome, n (%) |  |  | **0.007** |
| PD | 25 (7.1%) | 43 (12.3%) |  |
| SD | 10 (2.8%) | 19 (5.4%) |  |
| PR | 13 (3.7%) | 9 (2.6%) |  |
| CR | 131 (37.3%) | 101 (28.8%) |  |
| Age, median (IQR) | 69 (60, 76) | 68.5 (60, 76) | 0.908 |
