# Supplementary material for: Elevated ICAM5 as a promising predictor of poor prognosis in bladder cancer via EMT, immune microenvironment, and therapy resistance
Source: PLoS One. 2026 Jun 8;21(6):e0347623. doi: 10.1371/journal.pone.0347623 (PMC13245789; doi:10.1371/journal.pone.0347623)
Supplement: S2 Table — (DOCX) [file pone.0347623.s002.docx]

Supplementary table 2. The relationship between ICAM5 and clinical factors via single gene logistic model analysis in bladder cancer.

| Characteristics | Total(N) | Odds Ratio(OR) | P value |
| --- | --- | --- | --- |
| Gender (Female vs. Male) | 414 | 1.191 (0.769-1.849) | 0.435 |
| Age (>70 vs. <=70) | 414 | 1.040 (0.705-1.535) | 0.843 |
| T stage (T3&T4 vs. T1&T2) | 380 | 1.355 (0.882-2.088) | 0.167 |
| N stage (N1&N2&N3 vs. N0) | 370 | 1.280 (0.835-1.964) | 0.258 |
| M stage (M1 vs. M0) | 213 | 12.697 (2.366-235.202) | **0.016** |
| Pathologic stage (Stage III& IV vs. Stage I& II) | 412 | 1.447 (0.957-2.196) | 0.081 |
| Primary therapy outcome (PR&CR vs. PD&SD) | 357 | 0.393 (0.242-0.631) | **<0.001** |
| Histologic grade (High Grade vs. Low Grade) | 411 | 4.614 (1.672-16.254) | **0.007** |
| Subtype (Non-Papillary vs. Papillary) | 409 | 2.314 (1.517-3.561) | **<0.001** |
| Lymphovascular invasion (Yes vs. No) | 283 | 1.347 (0.844-2.158) | 0.213 |
