# Supplementary material for: Elevated ICAM5 as a promising predictor of poor prognosis in bladder cancer via EMT, immune microenvironment, and therapy resistance
Source: PLoS One. 2026 Jun 8;21(6):e0347623. doi: 10.1371/journal.pone.0347623 (PMC13245789; doi:10.1371/journal.pone.0347623)
Supplement: S3 Table — (DOCX) [file pone.0347623.s003.docx]

Supplementary table 3. Cox regression model analysis of overall survival in patient with bladder cancer.

| Characteristics | Total(N) | Univariate analysis | |  | Multivariate analysis | |
| --- | --- | --- | --- | --- | --- | --- |
|  |  | Hazard ratio (95% CI) | P value |  | Hazard ratio (95% CI) | P value |
| Age | 407 | 1.033 (1.017-1.049) | **<0.001** |  | 1.026 (1.000-1.054) | 0.054 |
| Gender | 407 |  |  |  |  |  |
| Female | 107 | Reference |  |  |  |  |
| Male | 300 | 0.874 (0.631-1.210) | 0.417 |  |  |  |
| T stage | 373 |  |  |  |  |  |
| T1&T2 | 122 | Reference |  |  |  |  |
| T3&T4 | 251 | 2.092 (1.439-3.041) | **<0.001** |  | 1.563 (0.473-5.157) | 0.464 |
| N stage | 365 |  |  |  |  |  |
| N0&N1 | 283 | Reference |  |  |  |  |
| N2&N3 | 82 | 2.240 (1.609-3.120) | **<0.001** |  | 1.733 (0.929-3.235) | 0.084 |
| M stage | 207 |  |  |  |  |  |
| M0 | 196 | Reference |  |  |  |  |
| M1 | 11 | 3.310 (1.582-6.926) | **0.001** |  | 1.105 (0.385-3.169) | 0.853 |
| Pathologic stage | 405 |  |  |  |  |  |
| Stage I&Stage II | 132 | Reference |  |  |  |  |
| Stage III&Stage IV | 273 | 2.203 (1.521-3.190) | **<0.001** |  | 1.127 (0.321-3.958) | 0.852 |
| Subtype | 402 |  |  |  |  |  |
| Non-Papillary | 271 | Reference |  |  |  |  |
| Papillary | 131 | 0.658 (0.462-0.938) | **0.021** |  | 0.833 (0.460-1.509) | 0.547 |
| Histologic grade | 404 |  |  |  |  |  |
| Low Grade | 21 | Reference |  |  |  |  |
| High Grade | 383 | 2.899 (0.717-11.718) | 0.135 |  |  |  |
| ICAM5 | 407 |  |  |  |  |  |
| Low | 204 | Reference |  |  |  |  |
| High | 203 | 1.417 (1.053-1.906) | **0.021** |  | 1.906 (1.127-3.223) | **0.016** |
