# Supplementary material for: Elevated ICAM5 as a promising predictor of poor prognosis in bladder cancer via EMT, immune microenvironment, and therapy resistance
Source: PLoS One. 2026 Jun 8;21(6):e0347623. doi: 10.1371/journal.pone.0347623 (PMC13245789; doi:10.1371/journal.pone.0347623)
Supplement: S1 Fig — (DOCX) [file pone.0347623.s004.docx]

**Western Blot Analysis**
The Western blot (WB) procedure was performed as previously described in our methodological references[1]. Briefly, total protein was extracted from cell lysates, separated by SDS-PAGE, and transferred to PVDF membranes. Membranes were probed with a primary antibody against ICAM5 (Proteintech, USA, Cat# 12759-1-AP) followed by incubation with an appropriate HRP-conjugated secondary antibody. Protein bands were visualized using enhanced chemiluminescence (ECL) and quantified with ImageJ software.


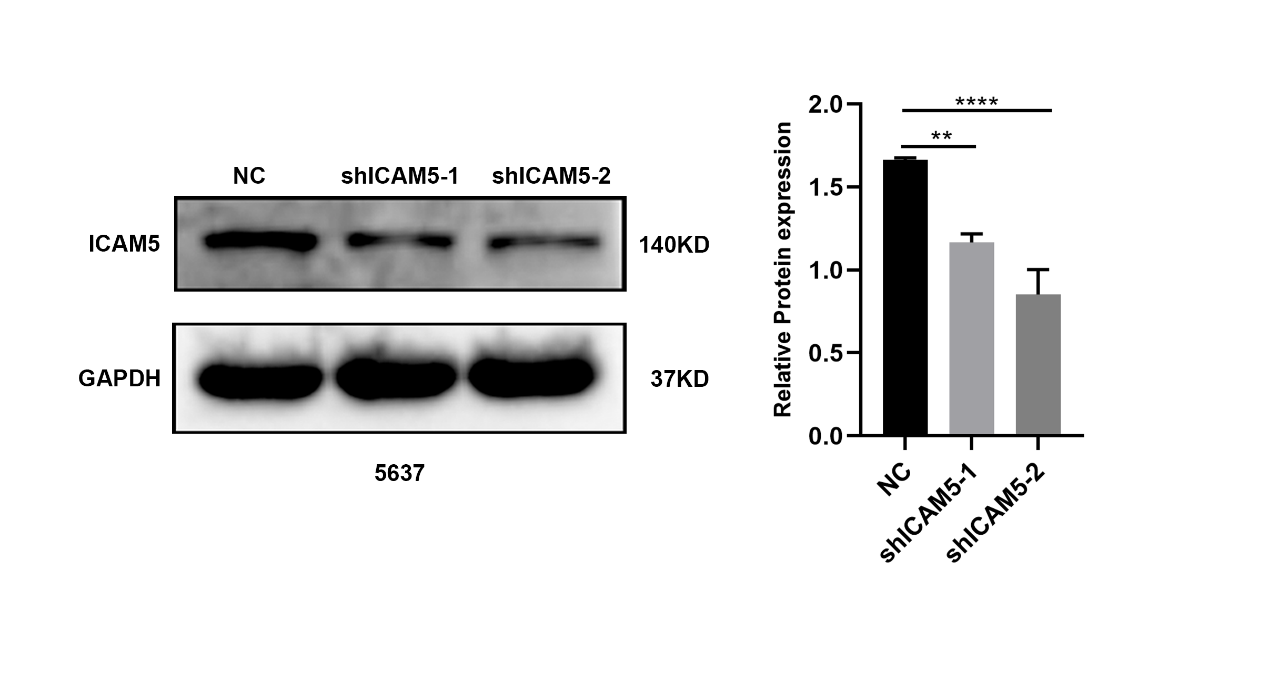


**Supplementary figure 1** Protein-Level Validation of ICAM5 Knockdown in 5637 Cells by Western Blot. (**p < 0.01, ****p < 0.0001.)

1. Chen X, Wang Q, Zhu Z, Peng Z, Huang K, Deng G, et al. TRIP13-induced NUSAP1 upregulation promotes CcRCC progression through EMT and PI3K/AKT/mTOR pathway. Journal of translational medicine. 2025;23(1):890. Epub 2025/08/12. doi: 10.1186/s12967-025-06761-3. PubMed PMID: 40790482; PubMed Central PMCID: PMCPMC12341202.
