# Supplementary figures and images for: Elevated ICAM5 as a promising predictor of poor prognosis in bladder cancer via EMT, immune microenvironment, and therapy resistance
Source: PLoS One. 2026 Jun 8;21(6):e0347623. doi: 10.1371/journal.pone.0347623 (PMC13245789; doi:10.1371/journal.pone.0347623)

**The raw data of Western Blot**

**
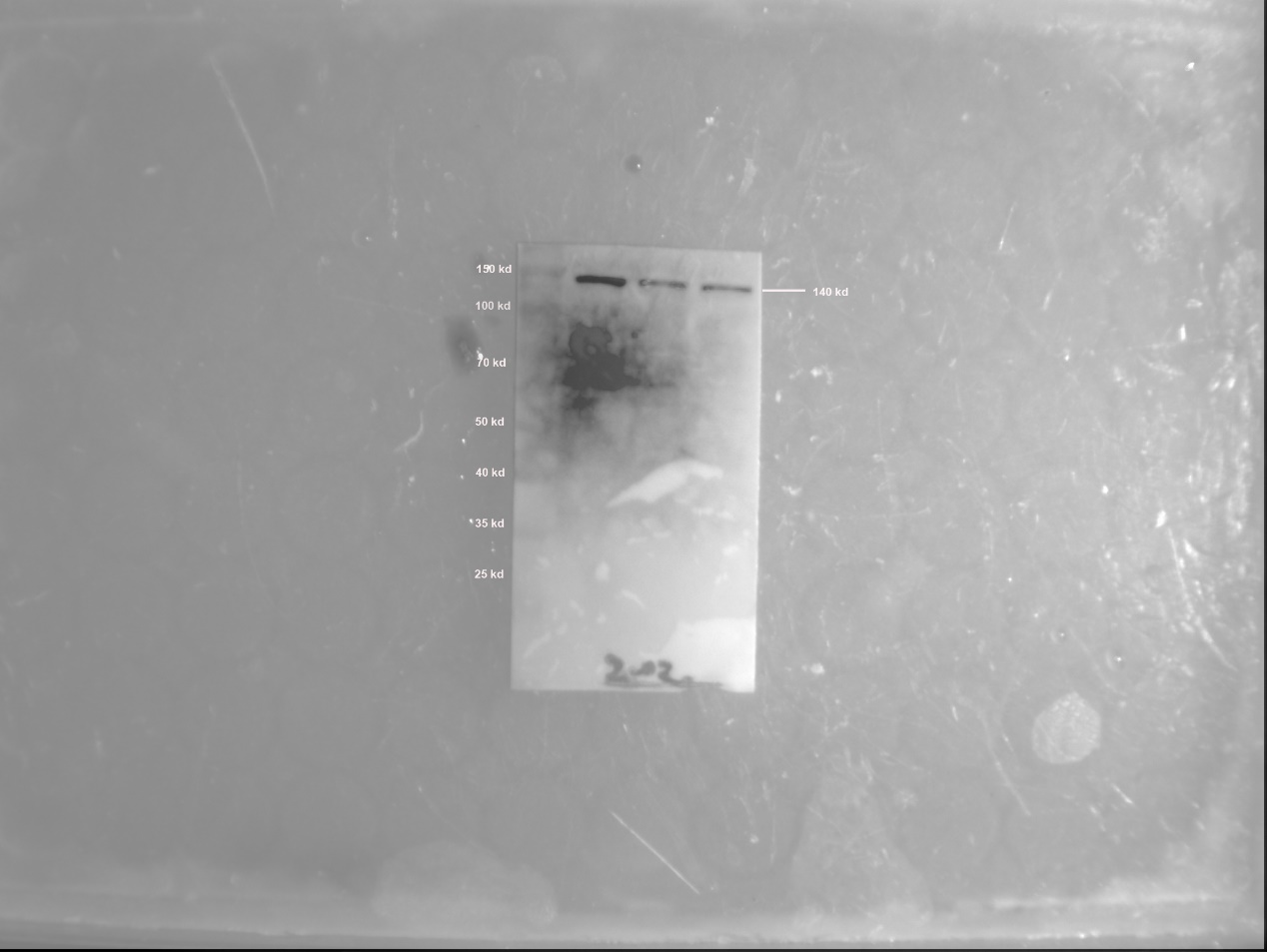
**


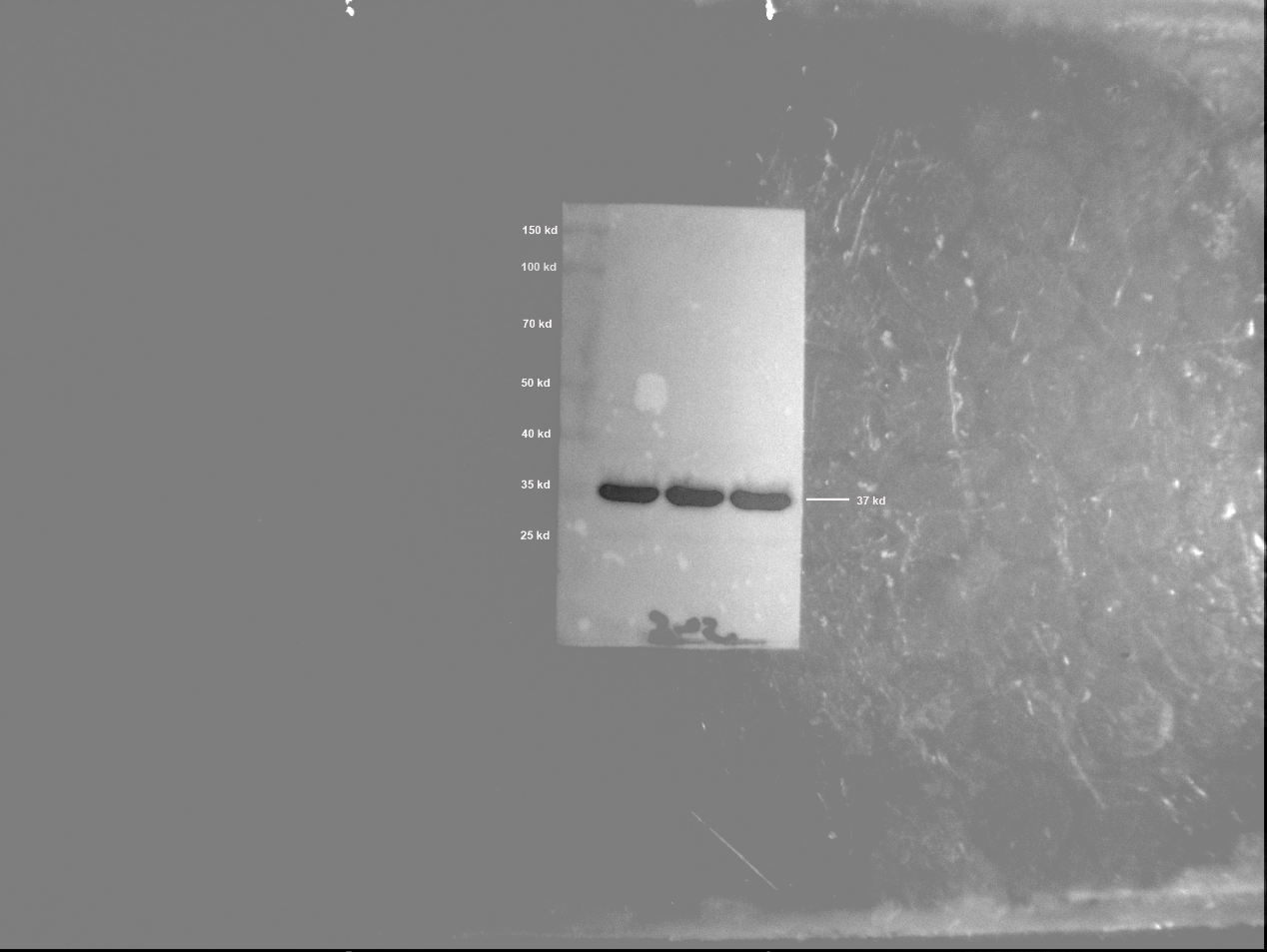


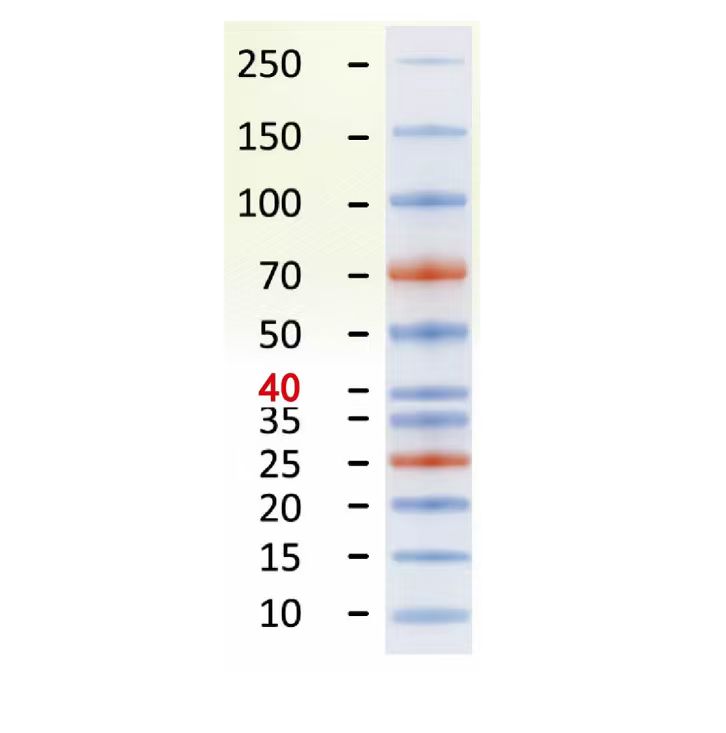

Supplement: S1 Data — (DOCX) [file pone.0347623.s005.docx]
